# Medicine

**Published:** 1904-03

**Authors:** Theodore Fisher


### Progress of the Medical Sciences.

### MEDICINE.

**Henoch's Purpura.**—A form of purpura occurring in children was described many years ago by Henoch, which, although not very uncommon, has not attracted much attention. Dr. Hale White has recently given a clinical lecture upon this disease,[1] illustrated by notes of two cases. The most characteristic

[1] *Guy's Hosp. Gaz.*, 1904, xviii. 44.

feature of the disease is the presence of abdominal pain of severe character, associated with vomiting and sometimes diarrhœa. Purpuric spots on the skin may precede or follow the abdominal pain. Sometimes there are hemorrhages from the mucous membranes, and hæmatemesis, passage of blood by the rectum, or hæmaturia may occur. As in some other forms of purpura, there may also be occasionally some pain and swelling of the joints. Another noteworthy feature is the liability of the purpura and other symptoms to recur, and Dr. Hale White refers to an interesting case in which a girl, aged 14, during a long period had one attack every week. It is scarcely necessary to remark, however, that recurrences at such short intervals must be extremely rare. Occasionally the vomiting may be so severe as to give rise to the idea that intestinal obstruction is present. Illustrations of this will be briefly referred to below. In passing, however, it may be mentioned that Mr. J. S. Judson[1] records a case in which stercoraceous vomiting was associated with purpura, thought to be rheumatic in origin. The patient was slightly older than the majority of those in which Henoch's purpura occurs. He was 17, and is said to have been ill with "rheumatism of subacute character" for ten days before the purpura appeared. At the time of admission to the District Infirmary, Ashton-under-Lyne, he was much collapsed with a quick, thready pulse. He complained of pains in the epigastric region and around the umbilicus, but there was no abdominal distension. A purpuric rash was present over the legs and forearms. The following day the vomit is said to have been "distinctly fæcal." The question of laparotomy was discussed but no operation was performed, and the symptoms subsided. If it were true rheumatism from which this boy suffered, and the purpura true rheumatic purpura, it is obvious that abdominal symptoms similar to those which occur in the disease known as Henoch's purpura may also arise in the course of peliosis rheumatica. The severity of the vomiting, however, in this case is the point of particular interest, especially as cases of Henoch's purpura have been recently described, in which intussusception was considered to be present.

G. A. Sutherland[2] reports an interesting example, but before noticing it in detail possibly the description by Dr. Sutherland of the symptoms referable to the abdomen present in Henoch's purpura may not be out of place. He says, "The abdominal pain is severe, of a colicky nature, and is referred to the umbilical, the epigastric, or the right inguinal regions. The patient cries out, tosses about, presses the hand on the abdomen, or presses the abdomen on the bed. Sickness soon follows of an exhausting nature, and frequently the vomited material is blood-stained or consists of pure blood. The tongue rapidly becomes coated, the breath is offensive, and all appetite is entirely lost.

[1] *Lancet*, 1903, ii. 824. [2] *Brit. J. Children's Dis.*, 1904, i. 23.

At first constipation is marked, but in a few days, with or without the employment of aperient medicine, diarrhœa sets in, the motions are offensive and blood-stained, and soon pure blood is passed—it may be in large quantity—from the bowels. The act of vomiting, or the evacuation of the bowels, seems to give temporary relief to the abdominal pain. The abdomen is usually retracted, tenderness may be present on pressure over the region of the colon, especially if tenesmus is a marked feature, but as a rule there is nothing abnormal to be detected on physical examination. Such an attack may last for a few hours or a few days, and then there is a gradual remission of the symptoms until the next attack."

The following are a few facts connected with a case under the care of Dr. Sutherland thought at one time possibly to be one of intussusception. A boy, aged 5 years, was admitted into the Paddington Green Children's Hospital for abdominal pain and vomiting. The bowels acted regularly at first, but then became blood-stained, and later pure blood was passed. The sickness ceased, but the abdominal pain was constant. At the time no purpuric spots were present. The abdomen was very resistant, and an examination was made under an anæsthetic, but nothing to account for the pain was found. The following day an operation was performed, and portions of intestine were discovered thickened by extravasated blood. For a few days after the operation the boy continued in a serious condition, but eventually the symptoms gradually subsided, and after a few recurrences of the "vomiting, purpura, melæna, abdominal pain, and extreme lethargy," eventually recovered, and left the hospital. It should be added that in this case purpuric spots appeared for the first time a few days after the operation had been performed.

A similar case is recorded in the same *Journal*[1] by Mr. Harold Burrows. A boy, aged 11 years, was admitted into the Bolingbroke Hospital, with a note from a doctor in which he was said to be suffering from "fæcal vomiting," and that he was "passing blood by the bowel" and "probably had an intussusception." The mother said that on the morning of the day of admission, after the boy had been "out of sorts" for some days, he was seized with "violent pains in the abdomen, and shortly afterwards passed a formed motion, with about a table-spoonful of blood, and was sick, the vomit being dark brown and smelling just like a motion." Mr. Burrows says that when he saw the boy he found him to be pale with an "abdominal" expression, and constantly moaning with pain which appeared to be intense, and was mainly localised in the epigastrium. The abdomen was nearly motionless, and the muscles were rigid, especially in the lower parts, and there was general tenderness all over the abdomen. The boy was anæsthetised, but nothing that threw any light upon the case

[1] *Brit. J. Children' Dis.*, 1904, i. 28.

was discovered. An exploratory laparotomy was therefore performed, but nothing found except some congested patches of small intestine. On the following day a careful search was made for petechiæ, and some small purpuric spots were found on the backs of the elbows, on the buttocks, and on each leg. For three weeks after the operation the boy appeared to be progressing favourably, except that there were frequent eruptions of purpuric spots. At the end of that time, however, he was again seized with severe abdominal pains, and passed shortly afterwards about 1½ ounces of blood from the bowels. "The pain was intense," but 15 minims of tincture of opium, given by the rectum brought some relief. Three days later there was a general eruption of purpura. After this second attack, however, he steadily improved, and when seen four months later appeared "the picture of health."

It may be interesting to note that a few years ago a somewhat similar case occurred in the Bristol Royal Infirmary, under the care of Dr. J. E. Shaw, which, however, ended, as Henoch's purpura very rarely ends, in death. The boy, aged 3½ years, was admitted with severe abdominal pain and passage of blood by the rectum, for supposed intussusception. He was examined under an anæsthetic, but nothing to account for the pain discovered. In his case an exploratory operation was not performed, and he left the Infirmary apparently well, to return, however, in three weeks with a recurrence of the symptoms which rapidly ended fatally. At the autopsy we found the walls of the whole length of the small intestine and of the ascending colon infiltrated with blood, and a large quantity of blood in the lumen. There were in this case no hemorrhages elsewhere in the body and no purpuric spots on the skin.

Dr. Raymond Crawfurd[1] has an article in which some points in connection with the subject of Henoch's purpura are referred to. His paper does not deal particularly with the unusually severe features of Henoch's purpura which we have been noticing, but, in passing, he mentions a case when he had known that "the diagnosis of acute intestinal obstruction was actually made until the appearance of the familiar symptoms cleared the picture." The special point upon which Dr. Crawfurd lays stress is that Henoch's purpura is scarcely a disease by itself, but is a form of erythema exudativum multiforme, in which abdominal pain is a marked symptom. He gives notes of three cases to illustrate his contention. Thus in a boy, aged 16, who sometimes had abdominal pain and vomiting associated with purpura, also suffered at other times from a non-purpuric erythematous rash. It is interesting to note that this boy sometimes had swelling of the joints with a rise of temperature associated with the purpuric attacks, an association that might, perhaps, lead it to be described as rheumatic purpura, a circumstance which reminds us of a case mentioned earlier in this

[1] *Lancet*, 1903, ii, 1154.

review. On the other hand, bleeding from the gums was also present—a form of hemorrhage which, it is needless to say, reminds us of scurvy.

It is not a new fact, however, that rashes other than purpuric spots may occur during the course of Henoch's purpura, and that some swelling of the joints may occur has frequently been noticed. It seems quite reasonable to suggest, as Dr. Crawfurd does, that Henoch's purpura is merely erythema exudativum multiforme, in which the abdominal features are especially well marked, but his cases seem to differ little from other recorded cases of the disease which bears the name of Henoch. Many erythemata are no doubt due to toxins absorbed from the alimentary canal, and Henoch's purpura, like these erythemata, is thought probably to be a result of such poisoning.

THEODORE FISHER.

